# Dynamics of organic matter and bacterial activity in the Fram Strait during summer and autumn

**DOI:** 10.1098/rsta.2019.0366

**Published:** 2020-08-31

**Authors:** Anabel von Jackowski, Julia Grosse, Eva-Maria Nöthig, Anja Engel

**Affiliations:** 1 GEOMAR Helmholz Centre for Ocean Research Kiel, Kiel, Germany; 2 Alfred Wegener Institute, Helmholtz Centre for Polar and Marine Research, Bremerhaven, Germany

**Keywords:** Arctic Ocean, seasonality, semi-labile organic carbon, bacteria

## Abstract

The Arctic Ocean is considerably affected by the consequences of global warming, including more extreme seasonal fluctuations in the physical environment. So far, little is known about seasonality in Arctic marine ecosystems in particular microbial dynamics and cycling of organic matter. The limited characterization can be partially attributed to logistic difficulties of sampling in the Arctic Ocean beyond the summer season. Here, we investigated the distribution and composition of dissolved organic matter (DOM), gel particles and heterotrophic bacterial activity in the Fram Strait during summer and autumn. Our results revealed that phytoplankton biomass influenced the concentration and composition of semi-labile dissolved organic carbon (DOC), which strongly decreased from summer to autumn. The seasonal decrease in bioavailability of DOM appeared to be the dominant control on bacterial abundance and activity, while no temperature effect was determined. Additionally, there were clear differences in transparent exopolymer particles (TEP) and Coomassie Blue stainable particles (CSP) dynamics. The amount of TEP and CSP decreased from summer to autumn, but CSP was relatively enriched in both seasons. Our study therewith indicates clear seasonal differences in the microbial cycling of organic matter in the Fram Strait. Our data may help to establish baseline knowledge about seasonal changes in microbial ecosystem dynamics to better assess the impact of environmental change in the warming Arctic Ocean.

This article is part of the theme issue ‘The changing Arctic Ocean: consequences for biological communities, biogeochemical processes and ecosystem functioning’.

## 1. Introduction

Anthropogenic climate change is warming the Arctic Ocean about two to three times faster than the global average [1]. A sensitive indicator of the degree of warming is the loss of sea ice. The year 2018 marked the sixth-lowest sea ice minimum in the nearly 40-year satellite record [2]. Warming of the Arctic results in seasonal, thin and fragile sea ice, thereby completely changing the landscape of icy ecosystems [3–6]. These environmental changes are already influencing phytoplankton dynamics since ice-retreat was responsible for the 30% increase of net primary production between 1998 and 2012 [7]. The change in phytoplankton dynamics could impact bacterial dynamics in the near future since phytoplankton release organic matter that is remineralized by bacteria. This remineralization drives the respiratory flux of CO_2_ from the ocean to the atmosphere. Despite the important role of bacteria in the global carbon cycle, the control on heterotrophic bacterial activity in the Arctic is not well-understood.

Recent studies have identified the lability of dissolved organic carbon (DOC) as a major factor controlling bacterial activity in polar environments [8–10]. The lability of DOC can be classified as a continuum from very labile to ultra-refractory DOC: labile components are used within hours to days, while refractory components have a residence time of centuries to millennia. Semi-labile DOC has a turnover of weeks to months, which allows it to accumulate in the upper water column during the productive season [11]. Dominant biochemical components within the semi-labile fraction include dissolved combined carbohydrates (dCCHO) and dissolved hydrolysable amino acids (dHAA) [12–14]. Furthermore, the semi-labile fraction can partition into gel particles, constituting a dynamic continuum from dissolved precursors to single colloids (approx. 1 nm) and macro gels (greater than 1 mm) [15–17]. Polysaccharide-containing micro gels, referred to as transparent exopolymer particles (TEP), are among the best-described micro gels in the ocean. The amount of TEP released into the ocean increases during the senescent bloom phase when phytoplankton growth approaches nutrient depletion [18–22]. Another type of micro gel are Coomassie Blue stainable particles (CSP). CSP are presumably formed by extracellular proteins, but few endeavours have explored their production [23,24]. The organic content that has aggregated as TEP and CSP can serve as substrates for particle-attached bacteria and also provide an important vector for export to the deep sea [25,26].

The degree to which microbial cycling of organic matter is subject to change under the pressure of global warming is difficult to predict. Satellite-based models have the advantage of using annual data and accounting for seasonal dynamics [7,27], but ecosystem or nutrient models rely on *in situ* data, like Forest *et al.* [28]. However, most *in situ* data in the Arctic are collected during the summer. This seasonal data bias is owing to the logistical difficulties of sampling in the Arctic during the dark and colder seasons. The predictions are further implicated by a regional bias since the majority of data for heterotrophic microbial processes are collected in the Beaufort Sea and Chukchi Sea [29]. Our study aimed at filling seasonal gaps by investigating DOM dynamics and bacterial activity in the Fram Strait during summer and autumn. The objective of this study was to assess (i) the seasonal shifts in DOC availability and in BP and (ii) to evaluate seasonal changes in DOM–microbe interactions for carbon-cycling within the pelagic Arctic ecosystem.

## 2. Methods

### (a) Study site

**Figure 1.**
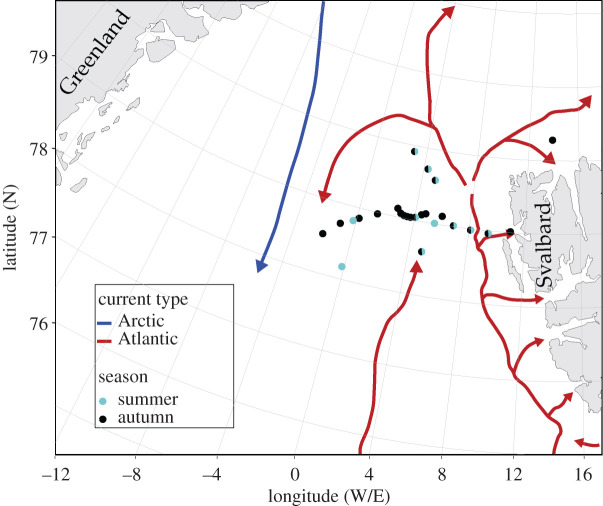
Map of stations within the Fram Strait in the proximity of the LTER observatory HAUSGARTEN. The samples during summer were collected with the RV *Polarstern* on board cruise PS114 from 16 July to 23 July 2018 and during autumn with the RV *Maria S. Merian* on board MSM77 from 16 September to 4 October 2018. Warm-water with Atlantic-origin (red arrows/northward) enters via the West Spitsbergen Current (WSC) in the eastern Fram Strait and cold polar water of Arctic-origin (blue arrows/southward) exits via East Greenland Current (EGC) in the west of the Fram Strait. (Online version in colour.)

Samples were collected in the Fram Strait with the RV *Polarstern* cruise PS114 from 16 to 23 July 2018 during summer and with the RV *Maria S. Merian* cruise MSM77 from 16 September to 4 October 2018 during autumn (figure 1; electronic supplementary material, table S1). The sampling campaigns were part of yearly measurements in proximity to the Long-Term Ecological Research (LTER) observatory HAUSGARTEN situated in the eastern Fram Strait. The Fram Strait is the only deep gateway to the central Arctic Ocean and is characterized by two distinct hydrographic regimes. In the east, the northward-flowing West Spitsbergen Current (WSC) transports warm saline Atlantic water (AW; >2°C; >34.9 PSU) into the Arctic basin. On the opposite side of the 450 km wide Strait, the southward-flowing East Greenland Current (EGC) transports cold polar water (PW; <0°C; <34.7 PSU) along the Greenland shelf. Here, water masses that were not distinctly characterized as AW or PW were defined as intermediate water (IW).

### (b) Discrete sampling parameters

Sampling procedures were identical during both cruises. Seawater samples were collected at five depths (surface; above, in, and below the deep chlorophyll maximum (DCM); 100 m) using a SEA-BIRD CTD rosette sampling system equipped with 24 Niskin bottles. The chlorophyll maximum was identified by running a fluorescence probe on the downward cast of the CTD.

Samples for chlorophyll-*a* were collected on 25 mm GF/F (Whatman, GE Healthcare Life Sciences, UK) and subsequently frozen (−20°C) until extraction using 90% acetone for photometric analyses (Turner Designs, USA), slightly modified after Evans *et al.* [30]. Chlorophyll-*a* was used as a proxy for phytoplankton biomass. Duplicate samples for dissolved organic carbon (DOC) and total dissolved nitrogen (TDN) were filtered through 0.45 μm GMF GD/X filters (Whatman, GE Healthcare Life Sciences, UK) and collected in combusted glass ampoules (8 h, 450°C). DOC/TDN was acidified and stored at 4°C until simultaneous analysis after Engel & Galgani [31] with a detection limit (DL) of 1 mol l^−1^. Duplicate samples for total dissolved phosphorus (TDP) were filtered through 0.45 μm Acrodisk filters (GHP membrane, Pall Corporation, USA) and frozen (−20°C) until analysis. TDP and dissolved inorganic phosphorus (DIP) were analysed after Murphy & Riley [32] with a DL of 2 μmol l^−1^. DIP was subtracted from TDP to obtain dissolved organic phosphorus (DOP). Duplicate samples for high-molecular-weight (greater than 1 kDa) dissolved combined carbohydrates (dCCHO) were filtered through 0.45 μm Acrodisk filters, collected in combusted glass vials (8 h, 450°C) and frozen (−20°C) until analysis after Engel & Händel [33] with a DL of 10 nmol l^−1^. The analysis classified 11 monomers: arabinose, fucose, galactose, galactosamine, galacturonic acid, glucosamine, glucose, glucuronic acid, rhamnose, co-elute mannose and xylose. Duplicate samples for dissolved hydrolysable amino acids (dHAA) were filtered through 0.45 μm Acrodisk filters, collected in combusted glass vials (8 h, 450°C) and frozen (−20°C) until analysis. dHAA were measured with ortho-phthaldialdehyde derivatization by high-performance liquid chromatography (HPLC) [34,35]. The HPLC (Agilent Technologies, USA) was equipped with a C18 column (Phenomenex, USA) with a precision of less than 5% and DL of 2 nmol l^−1^. The analysis classified 13 monomers: alanine, arginine, aspartic acid, isoleucine, glutamic acid, glycine, leucine, phenylalanine, serine, threonine, tyrosine, valine; and *γ*-aminobutyric acid (GABA).

Duplicate samples for gel particle analysis were filtered onto 25 mm 0.4 μm-pore sized Nucleopore track-etched polycarbonate filters (Whatman, GE Healthcare Life Sciences, UK). Filters for transparent exopolymer particle (TEP) analysis were stained using Alcian Blue [36] for 5 s, whereas those for Coomassie Blue stainable particles (CSP) were stained with Coomassie Blue [23] for 30 s. Filters were subsequently placed on CytoClear slides (Poretics Inc., USA) and then frozen (−20°C) until analysis. Slides were imaged using an Axioscope A.1 with an attached Axio-Cam MRC (Zeiss, Germany) at 20× magnification and processed with ImageJ [22]. The image processing allowed the determination of particle abundance as well as particle size (area). Particle abundance is of interest for aggregation and degradation processes [37], whereas the size is a measure for estimating the carbon and nitrogen content of the individual gels [38,39].

Samples for cell abundance were fixed with glutardialdehyde (GDA, 25%), and subsequently frozen (−80°C) until further analysis. Prior to analysis, the flow cytometer (FACSCalibur, Becton Dickinson, USA) was calibrated and standardized with TruCount beads (Becton Dickinson, USA). The cells were stained using SybrGreen I (Thermo Fisher Scientific, USA) and subsequently counted by flow cytometry using the Cell Quest 3.3 software with a DL of 2000 events per second. Additionally, subpopulations of low nucleic acid content (LNA) and high nucleic acid content (HNA) bacteria were derived by distinguishing between differences in fluorescence intensity [40–42].

Bacterial production (BP) was measured onboard the research vessels using the microcentrifuge method [43]. Duplicate samples and one killed control (1.5 ml each) were labelled using ^3^H-leucine (BioTrend, USA) at a final concentration of 20 nmol l^−1^. ^3^H-leucine had a specific activity of 100Ci mmol^−1^. The samples were incubated for 6 h in the dark at 4°C and terminated using trichloroacetic acid (TCA) at a final concentration of 5%. Leucine incorporation was converted into BP by applying a factor of 1.5 kg C mol leucine^−1^ assuming no intracellular isotope dilution [44,45].

### (c) Calculations and statistical analysis

In the text and tables, *n* refers to the number of observations and *n*_st_ to the number of integrated stations. Stations were integrated over the upper 100 m using trapezoidal integration. The calculations for the carbon and nitrogen content of dCCHO and dHAA were based on carbon and nitrogen atoms contained in the identified monomers. Carbon content of dCCHO and dHAA was normalized to DOC as % DOC. The incorporation of semi-labile DOC into BP per day (% SL-DOC d^−1^) was calculated by BP (mmol C m^−2^ d^−1^) ÷ semi-labile DOC (mmol m^−2^) × 100.

Statistical analyses were conducted using the software R (v3.5.1) in Rstudio (v1.1.414; [46]). Variables were fed into a statistical mixed model [47,48] including season (‘Summer’,‘Autumn’) and either depth (‘surface’, ‘above DCM’, ‘DCM’, ‘below DCM’, ‘100 m’) or water mass (‘AW’, ‘IW’, ‘PW’) as well as their interaction term as fixed factors. The station was regarded as a random factor and the residuals were assumed to be normally distributed and to be homo/heteroscedastic with respect to the different levels. Based on this model, a pseudo-*r*^2^ [49] and an analysis of variances (ANOVA) was conducted, followed by multiple contrast tests (MCT) in order to compare the several levels of the influence factors, respectively. Statistical results are reported in electronic supplementary material, table S2.

Packages used in the scope of this study included PlotSvalbard (v0.7.11, [50]), ggplot2 (v3.2.0, [51]), nlme (v.3.1-139 [52]), piecewiseSEM (v2.0.2 [53]), multcomp (v1.4-10 [54]), lsmeans (v2.30-0 [55]), car(v3.0-2 [56]), FactorMineR (v1.41 [57]) and corrgram (v1.13 [58]).

## 3. Results

**Figure 2.**
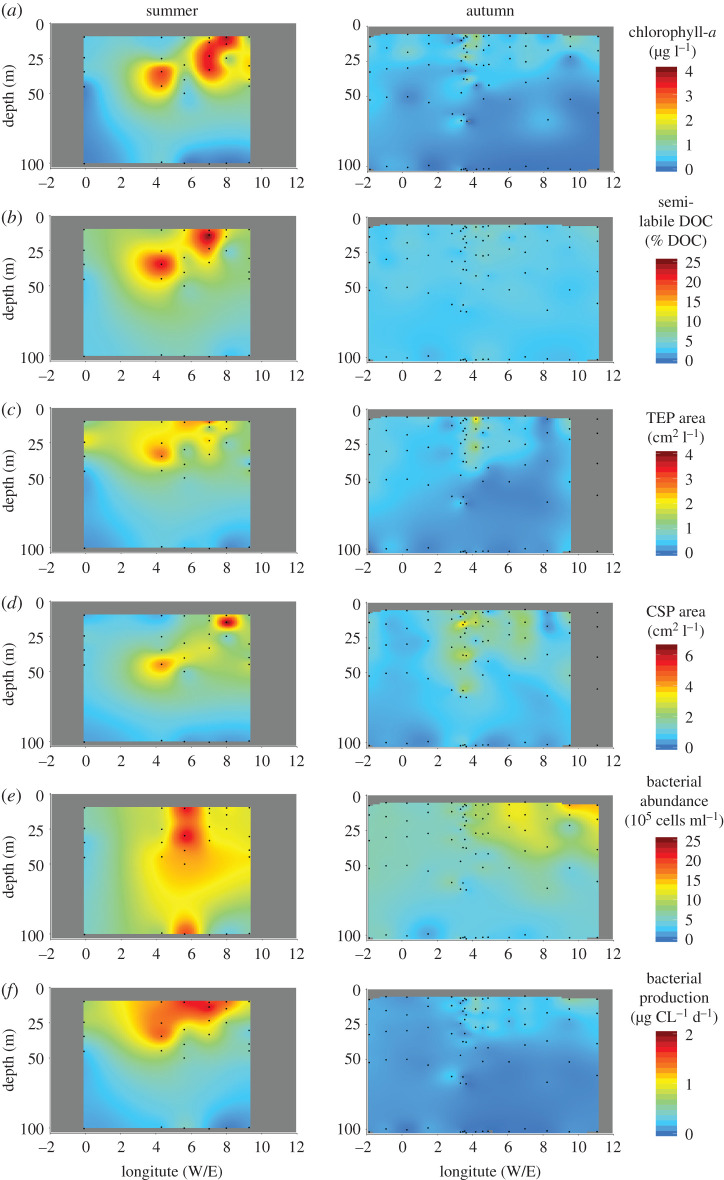
Spatial variability along the approximately 79°N latitude in the Fram Strait during the summer (left) and autumn (right) of 2018. The parameters shown include (*a*) chlorophyll-*a*, (*b*) semi-labile carbon, area of (*c*) TEP and (*d*) CSP, (*e*) bacterial abundance and (*f* ) bacterial production (BP). No data are available for the grey shaded region; stations not shown include: N3-N5, NSB1, S3, R1 (electronic supplementary material, table S1). (Online version in colour.)

The study area was predominantly ice-free during both summer and autumn. Ice floes were observed north of 79.5°N and west of 0°W/E in summer and north of 79.5°N and further west at 2°W in autumn. Single ice floes were encountered at ‘N4’ in summer and ‘D4’ in autumn (electronic supplementary material, table S1). Water temperature ranged from 0.75°C to 6.2°C in summer (*n* = 53) and from 0.52°C to 7.1°C in autumn (*n* = 110; table 1), therefore only characterizing as AT and IW. During both cruises, a fluorescence peak was observed between 20 and 40 m water depth indicating a DCM. However, the fluorescence peak was of lower intensity and occasionally below the detection limit at some stations in autumn. Chlorophyll-*a* ranged from 21 to 209 mg m^−2^ in summer (*n*_st_ = 11) and from 15 to 41 mg m^−2^ in autumn (*n*_st_ = 22) and thus was three times higher in summer compared to autumn (figure 2, table 1). Chlorophyll-*a* concentrations were significantly different in the AW between the summer and autumn (ANOVA *F*_1,136_ = 39.4, *p* < 0.0001; MCT *p* < 0.001; electronic supplementary material, table S2), while concentrations did not significantly change in IW.

**Table 1.** Arithmetic means and one standard deviation of variables determined in the Fram Strait during summer and autumn. The ‘*n*’ refers to the number of observations.

		summer	*n*	autumn	*n*
temperature	°C	4.50 ± 1.49	53	4.59 ± 1.52	110
salinity	PSU	34.84 ± 0.40	53	34.82 ± 0.29	110
chlorophyll-*a*	μg l^−1^	1.11 ± 1.02	53	0.36 ± 0.29	110
DOC	μmol l^−1^	73.65 ± 9.04	53	72.46 ± 5.64	110
semi-labile DOC	μmol l^−1^	6.24 ± 3.49	53	2.52 ± 0.60	104
semi-labile DOC	% DOC	8.4 ± 4.7	53	3.5 ± 0.8	104
dCCHO	μmol C l^−1^	4.65 ± 2.68	53	1.87 ± 0.47	110
dCCHO	% DOC	6.3 ± 3.6	53	2.6 ± 0.7	110
dHAA	μmol C l^−1^	1.59 ± 0.93	53	0.66 ± 0.20	104
dHAA	% DOC	2.1 ± 1.2	53	1.0 ± 0.3	104
semi-labile C : N	ratio	10.7 ± 2.0	53	9.3 ± 1.5	104
TEP particles	×10^6^ l^−1^	11.58 ± 8.17	53	7.01 ± 4.80	95
TEP area	cm^2^ l^−1^	1.08 ± 0.71	53	0.42 ± 0.29	95
TEP	μg C l^−1^	21.4 ± 14.5	53	7.1 ± 5.2	95
CSP particles	×10^6^ l^−1^	14.64 ± 9.38	53	11.69 ± 7.92	95
CSP area	cm^2^ l^−1^	1.43 ± 1.16	53	1.06 ± 0.74	95
bacterial abundance	×10^5^ ml^−1^	9.35 ± 4.36	53	6.41 ± 2.65	110
HNA	%	60.2 ± 7.8	53	50.7 ± 5.5	110
BP	μg C l^−1^ d^−1^	0.65 ± 0.47	52	0.14 ± 0.11	110
BP_cell_	fg C cell^−1^ d^−1^	0.71 ± 0.43	52	0.21 ± 0.11	110

### (a) Composition of organic matter

**Figure 3.**
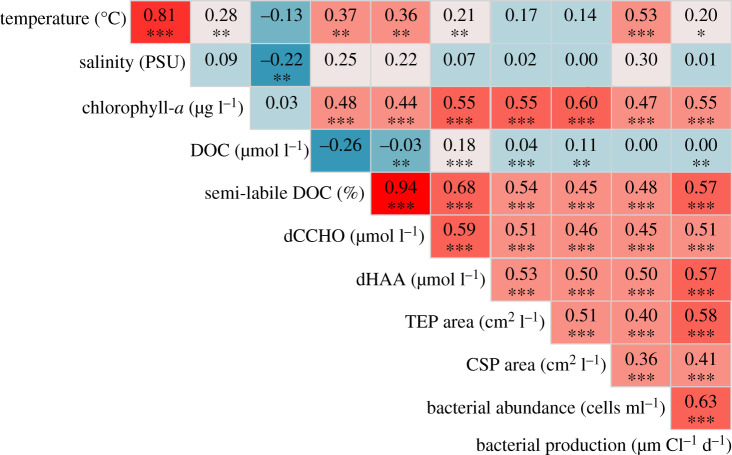
Correlations of physical and biochemical parameters in the upper 100 m of the Fram Strait in 2018. Low correlations are shown in a blue shade, followed by teal, grey, salmon and red indicating a strong correlation. Significances are shown by asterisks as ‘***’ <0.001, ‘**’ <0.01, ‘*’ <0.05 and ‘ ’ >0.05. (Online version in colour.)

**Figure 4.**
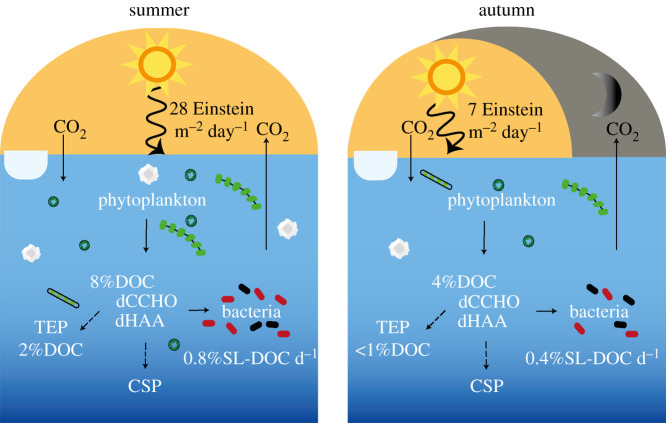
Partitioning of organic carbon between the dissolved and microbial pools for integrated water column of the Fram Strait during summer (left) and autumn (right). Phytoplankton release semi-labile DOM components like dCCHO and dHAA that can further partition into TEP and CSP. The decline in semi-labile DOC triggers a decrease in bacterial incorporation of semi-labile DOC (%SL-DOC d^−1^) and the bacterial community composition from summer to autumn. HNA bacteria are coloured in red and LNA bacteria in black. PAR data are a monthly estimate from MODIS-Aqua satellite of a 4-km spatial resolution between 1–31 July 2018 for summer and 1–30 September 2018 within $78-80^{\circ }\text{N}$ and −2°W–13°E from https://giovanni.gsfc.nasa.gov. (Online version in colour.)

DOC ranged from 6029 to 8171 mmol m^−2^ in summer (*n*_st_ = 11) and from 5953 to 8103 mmol m^−2^ in autumn (*n*_st_ = 22). DOC concentrations (table 1) showed no significant seasonal difference (*Mann–Whitney*-test, *p* = 0.8; electronic supplementary material, table S2). Semi-labile DOC made up 8.4 ± 4.7% of total DOC (% DOC) in summer (*n*=53) and 3.5 ± 0.8% DOC in autumn (*n* = 110, figures 2 and 4). Semi-labile DOC, and components thereof, significantly correlated with chlorophyll-*a* concentrations (figure 3). Furthermore, semi-labile DOC was significantly different between AW and IW in summer, but not autumn (ANOVA *F*_1,130_ = 6.1, *p* < 0.05; MCT *p* < 0.001; electronic supplementary material, table S2). TDN ranged from 819 to 1287 mmol m^−2^ in summer (*n*_st_ = 11) and from 780 to 1597 mmol m^−2^ in autumn (*n*_st_ = 22). TDP ranged from 31 to 49 mmol m^−2^ in summer (*n*_st_ = 11) and from 38 to 121 mmol m^−2^ in autumn (*n*_st_ = 22). DOP ranged from 5 to 21 mmol m^−2^ in summer (*n*_st_ = 11) and from 2 to 11 mmol m^−2^ in autumn (*n*_st_ = 9).

dCCHO ranged from 43 to 107 mmol m^−2^ in summer (*n*_st_ = 11) and from 24 to 42 mmol m^−2^ in autumn (*n*_st_ = 22). The molecular composition shows that dCCHO was dominated by glucose (14–66 mol%) and co-elute mannose/xylose (14–55 mol%) during both seasons. To investigate a potential change in dCCHO composition along with the quantitative decrease from summer to autumn, we applied principal component analysis (PCA). The first principal component (PC1) explained 51.9% and was primarily influenced by the season (electronic supplementary material, figure S1). The second principal component (PC2) explained 17.7% and was primarily influenced by the depth (electronic supplementary material, figure S1). dCCHO was significantly different between summer and autumn (*Mann–Whitney*-test, *p* < 0.0001) in all depths (ANOVA *F*_4,130_ = 17.4, *p* < 0.0001; MCT *p* < 0.01; electronic supplementary material, table S2). dCCHO contributed 6.3 ± 3.6% DOC to semi-labile DOC in summer (*n*=53) and 2.6 ± 0.7% DOC in autumn (*n* = 110, table 1).

dHAA ranged from 22 to 62 mmol m^−2^ in summer (*n*_st_ = 11) and from 13 to 20 mmol m^−2^ in autumn (*n*_st_ = 19). The molecular composition showed that dHAA was dominated by glycine in summer (13–35 mol%; 23.6 ± 4.8 mol%; *n* = 53) and autumn (23–38 mol%; 30.8 ± 2.8 mol%; *n*=104). The second most abundant dHAA was glutamic acid, which was higher in summer (8–24 mol%; 15.1 ± 3.7 mol%; *n* = 53) than in autumn (7–17 mol%; 10.5 ± 1.7 mol%; *n* = 104). Again, we applied PCA using the relative composition to determine a change in quality and degradation state [59]. PC1 explained 40.1% and was primarily influenced by the depth (electronic supplementary material, figure S2). PC2 explained 12.3% and was primarily influenced by the season (electronic supplementary material, figure S2). There was a significant difference between summer and autumn (*Mann–Whitney*-test, *p* < 0.0001) by depth and water masses (electronic supplementary material, table S2). dHAA contributed 2.1 ± 1.2% DOC in summer (*n* = 53) and 1.0 ± 0.3% DOC in autumn (*n* = 104, table 1).

TEP abundance ranged from 365 to 1936 × 10^6^ m^−2^ in summer (*n*_st_ = 11) and from 220 to 1398 × 10^6^ m^−2^ in autumn (*n*_st_ = 18). The total area of TEP ranged from 45 to 295 × 10^6^ cm^2^ m^−2^ in summer (*n*_st_ = 11) and from 4 to 12 × 10^4^ cm^2^ m^−2^ in autumn (*n*_st_ = 19). The area of TEP was significantly different between summer and autumn (*Mann–Whitney*-test; *p* < 0.0001; electronic supplementary material, table S2) with the strongest differences in the surface to below DCM (ANOVA *F*_4,118_ = 16.0, *p* < 0.0001; MCT *p* < 0.0001; electronic supplementary material, table S2). Concentration of carbon in TEP (TEP-C) ranged from 82 to 255 mmol m^−2^ in summer (*n*_st_ = 11) and to 19 to 115 mmol m^−2^ in autumn (*n*_st_ = 19). TEP-C relative to the DOC pool was 2.1 ± 0.9% DOC (*n*_st_ = 11) in summer and 0.7 ± 0.3% DOC in autumn (*n*_st_ = 19; figure 4). CSP abundance ranged from 672 to 1801 × 10^6^ m^−2^ in summer (*n*_st_ = 11) and from 284 to 2344 × 10^6^ m^−2^ in autumn (*n*_st_ = 19). The total area of CSP ranged from 63 to 377 × 10^6^ cm^2^ m^−2^ in summer (*n*_st_ = 11) and from 3 to 25 × 10^4^ cm^2^ m^−2^ in autumn (*n*_st_ = 19). The [TEP] : [CSP] ratio (area : area) was 1 : 1.6 in summer and 1 : 3.0 in autumn, reflecting the relative increase of CSP in autumn.

### (b) Bacterial distribution and production

Bacterial abundance ranged from 3 to $22\times 10^{5}\;{\text{cells ml}}^{-1}$ in summer (*n* = 53) and from 2 to $15\times 10^{5}\;{\text{cells ml}}^{-1}$ in autumn (*n* = 110, figure 2). Bacterial abundance was significantly different in AW, but not IW between summer and autumn showing that bacteria behave differently in the two water masses (ANOVA *F*_1,136_ = 10.6 *p* < 0.001; MCT; electronic supplementary material, table S2). Bacterial abundances in AW decreased from $10.34\pm 4.32\times 10^{5}\;{\text{cells ml}}^{-1}$ in summer (*n* = 41) to $6.02\pm 2.53\times 10^{5}\;{\text{cells ml}}^{-1}$ in autumn (*n* = 59), compared to the IW where it increased from $5.97\pm 2.40\times 10^{5}\;{\text{cells ml}}^{-1}$ in summer (*n* = 11) to $6.87\pm 2.73\times 10^{5}\;{\text{cells ml}}^{-1}$ in autumn (*n* = 51). Yet, the significant correlations with chlorophyll-*a* (*r*^2^ = 0.47, *p* < 0.001) and semi-labile DOC (*r*^2^ = 0.48, *p* < 0.001, figure 3), indicate that semi-labile DOC controlled bacterial abundances in all water masses. In addition, we differentiated between LNA and HNA. Abundance of LNA ranged from 1 to $9\times 10^{5}\;{\text{cells ml}}^{-1}$ in summer (*n* = 53) and 1 to $8\times 10^{5}\;{\text{cells ml}}^{-1}$ in autumn (*n* = 110). The abundance of HNA ranged from 2 to $13\times 10^{5}\;{\text{cells ml}}^{-1}$ in summer (*n* = 53) and 1 to $9\times 10^{5}\;{\text{cells ml}}^{-1}$ in autumn (*n* = 110). Therefore, the fraction of HNA decreased from summer to autumn (figure 4, table 1). The ratio of [HNA] : [LNA] (abundance : abundance) was 1.6 : 1 in summer (*n* = 53) and 1.1 : 1 in autumn (*n* = 110).

BP was measured at 4°C for all samples and ranged from 15 to 75 mg C m^−2^ d^−1^ in summer (*n*_st_ = 11) and from 3 to 15 mg C m^−2^ d^−1^ in autumn (*n*_st_ = 22). BP was more than four times higher in summer than in autumn (table 1) and significantly changed in all depths (ANOVA *F*_4,129_ = 16.9 *p* < 0.001; electronic supplementary material, table S2). Again, the significant correlation to chlorophyll-*a* as well as labile components (figure 3) suggests that the lability was responsible for the significant difference in BP between summer and autumn. Carbon incorporated into BP was 0.80 ± $0.37\text{\%}\;\text{SL-DOC}\;{\text{d}}^{-1}$ in summer (*n* = 11) and $0.45\pm 0.27\text{\%}\;{\text{SL-DOC d}}^{-1}$ in autumn (*n*=22; figure 4).

## 4. Discussion

Our study focuses on potential changes in DOM composition and the dependency of microbial activity in the Fram Strait between summer and autumn. Conditions during the field sampling in summer reflected the late-bloom phase of the main annual phytoplankton bloom development, which typically occurs in June/July [60]. Phytoplankton release an increased amount of bioavailable DOM towards the end of the bloom [33,61], which can explain the observed twofold higher concentration of dCCHO and dHAA in summer compared to autumn. The observed concentrations of 0.80 ± 0.46 μmol dCCHO l^−1^ in the Fram Strait are close to the 0.90 ± 0.32 μmol dCCHO l^−1^ reported for the Beaufort Sea during summer [62]. Accordingly, the average contribution of dCCHO to the DOC pool (% DOC) of 6.3 ± 3.6% DOC is lower than the 8 ± 3% DOC in Beaufort Basin [62]. In addition to the spatial variations, carbohydrates displayed a temporal variation. To the best of our knowledge, this is the first study showing a twofold decline of dCCHO between summer and autumn for the Arctic. The observed change is similar to the decline from approximately 4% DOC in summer to approximately 2% DOC in winter shown for the euphotic zone of the Sargasso Sea [13]. Furthermore, the decrease by 3.7% DOC until autumn resembles the trend between the upper 80 m and the deep Beaufort basin in summer (3% DOC [62]). This indicates that the seasonal production of bioavailable DOM within the Fram Strait was almost degraded until autumn. The remaining carbohydrates in the water column exhibited a more refractory state but could be a potential substrate for bacteria during the winter. Although carbohydrates are an important carbon and energy source for bacteria, amino acids also serve as a nitrogen source and directly support biomass production [63–69]. The seasonal trajectory of dHAA appears to increase from 1.5% DOC in spring [64] to 2.1–2.3% DOC in summer ([64], this study) and subsequently decreases to 1.0 ± 0.3% DOC until autumn. In particular, the drawdown of dHAA below the 1.1% DOC, a threshold set for the least reactive fraction of semi-labile DOM using bioassay experiments [65], indicates that the labile dHAA reservoir is likely to be exhausted in autumn. Changes in the seasonal dHAA reservoir are furthermore apparent in the amino acid composition (electronic supplementary material, figure S2). For example, the molar fraction of glutamic acid increased by 4.7 mol% between spring and summer [64], whereas it decreased by 4.6 mol% between summer and autumn in this study. In contrast, glycine decreased between spring and summer by 2.3 mol% [64], while it increased between summer and autumn by 7.5 mol% in this study. Consequently, the amino acid reservoir appears to be highly dynamic throughout the year with an increase of semi-labile components toward summer and a rapid utilization until autumn.

BP persists in different magnitudes throughout all seasons in the Arctic, albeit with a strong decrease from the productive to the unproductive season [70–75]. Measured BP rates determined for summer during this study are representative for the Fram Strait, i.e. surface BP 1.12 ± 0.4 μg C l^−1^ d^−1^ is very close to 1.14 ± 0.4 μg C l^−1^ d^−1^ observed between $0.8-9.5^{\circ }\text{E}$ in 2011 [60]. The fourfold decline observed from summer to autumn is similar to the decreases previously reported for coastal regions such as Franklin Bay, Canada [70,72] and Kongsfjorden, Svalbard [71]. In addition to BP, the bacterial abundance and the proportion of HNA declined from summer to autumn. HNA are considered to represent the more metabolically active subpopulation that also appears to be substrate-driven [60,76]. Consequently, as semi-labile DOC components declined so did BP and the proportion of HNA, which suggests that bacteria are primarily controlled by the availability of semi-labile DOC in summer and autumn (figure 4). However, semi-labile DOC can be equally as important as temperature in controlling bacterial activity [10,45,77,78]. Our study does not directly support this since temperatures remained unchanged and BP still declined from summer to autumn, but synergistic combined effects could arise during other seasons or in the future due to warming. Synergistic effects have been observed if the demand for labile organic matter is met [10]. When future scenarios of climate warming are taken into account, an elevated release of semi-labile DOC in summer could lead to an increase of BP under warmer temperatures. The synergistic effects may be less pronounced in autumn and winter when bacteria consume organic matter with a more refractory state. Alternatively, a rise in temperature might also allow microbial degradation of refractory compounds [79], indicating that the reactivity of DOC could be less controlling for BP in the future. At the time of our study, the bioavailability of DOM has been the dominant control on bacterial activity during the productive and unproductive season. This, however, does not rule out the possibility of synergistic effects in the future.

Bacteria might further supplement their demand for labile components by accessing the dynamic continuum of gel particles. Precursors of gel particles like TEP are released by phytoplankton during the senescent bloom phase [21,80,81]. In particular, the inter-annual variability of TEP in the Fram Strait has been related to the abundance of the prymnesiophyte *Phaeocystis pouchetii* [80]. The twofold higher phytoplankton biomass and amounts of TEP in our study compared to previous observations by Busch *et al.* [82] suggests that the phytoplankton community release different concentrations of TEP into the water column during summer. To the best of our knowledge, our study is the first to show that TEP abundance, area and carbon content decreased more than twofold from summer to autumn (figures 2 and 4). The decrease in abundance and carbon content of TEP indicates that the particle content attributed to *P. pouchettii* diminishes quickly over time [83]. Within a given season, TEP could have been subject to microbial remineralization by extracellular enzymes [81,84], coagulation processes and sinking into the deeper water column [82]. In contrast to TEP, CSP abundance and area declined by a factor less than one between the two seasons (figure 2, table 1). The production of CSP has been related to the picocyanobacterium *Synechococcus* spp. [85], which is highly abundant in the Arctic gateway throughout the year [86]. Again, amounts of CSP in summer and autumn were twice as high compared to 2015 [82], suggesting that formation by phytoplankton precursors, cell breakage and lysis [25] was higher during our study. Therefore, our results imply that cell deaths from the diatom-dominated summer bloom and the production by *Synechococcus* continue to release CSP until autumn. The continued amounts of CSP provide protein-rich micro gels for bacterial degradation after the dissolved pool of dHAA has been used. Our results show the loss of TEP and relative enrichment of CSP from summer to autumn thereby suggesting that gel particles could provide different temporal functions to the microbial food web. Future investigations of gel particle composition and the associated community composition might resolve whether the two types of particles provide different micro-niches for particle-attached bacteria at different times of the year [87–89].

Microbial dynamics within the Arctic ecosystem are seasonally and regionally variable, which makes an assessment of future changes challenging. Climate change associated alterations, such as rising temperatures and decreasing nutrient budgets, hold the potential to support phytoplankton release of semi-labile DOC that could support higher BP rates in summer and the beginning of autumn. Unfortunately, the lack of data for autumn 2018 did not allow us to discuss nutrients in the scope of this study. In the future, an increase in the amount of bioavailable DOM could stimulate the competition for mineral nutrients between phytoplankton and bacteria [90], releasing more CO_2_ during bacterial respiration. Therefore, future changes to BP might reduce the net community production in the microbial food web and weaken CO_2_ sequestration in the Arctic [27]. As such, parameters characterizing microbial food web dynamics, including components within semi-labile DOM, are of importance if we aim to assess future carbon cycling in the changing Arctic ecosystem.

## 5. Conclusion

Our observations demonstrate the importance of studying the effect of seasonal DOM dynamics on the microbial food web. Sampling in summer and autumn has allowed us to evaluate part of the seasonal shifts in microbial cycling of organic matter within the Fram Strait. Elevated concentrations of semi-labile DOC indicate an accumulation of recently produced DOM in summer. In autumn, the decrease in semi-labile DOC coincided with a decline in bacterial abundances and BP. Furthermore, we observed clear differences in the seasonal progression of gel particles with a twofold decrease in TEP and relative enrichment in CSP between summer and autumn. Our study highlighted that the availability of DOM shifts from summer to autumn and controls DOM–microbe interactions in the Fram Strait. Understanding the seasonal shifts in microbial cycling is of great importance in vulnerable environments like the Arctic, since seasonality may change in the future and potentially weaken the CO_2_ sequestration in the Arctic Ocean.

## Supplementary Material

Figure S1;Figure S2;Table S1;Table S2

## Data accessibility

Data are available on PANGAEA Database [91,92].
