# Supplementary material for: Dynamics of organic matter and bacterial activity in the Fram Strait during summer and autumn
Source: Philos Trans A Math Phys Eng Sci. 2020 Aug 31;378(2181):20190366. doi: 10.1098/rsta.2019.0366 (PMC7481659; doi:10.1098/rsta.2019.0366)
Supplement: Figure S1;Figure S2;Table S1;Table S2 [file rsta20190366supp1.pdf]

6. Supplementary Figures & Tables

Dynamics of organic matter and bacterial activity in the Fram Strait during summer and autumn

Anabel von Jackowski[1], Julia Grosse[1], Eva-Maria Nöthig[2] and Anja Engel[1]

[1]GEOMAR Helmholz Centre for Ocean Research, Kiel, Germany
[2]Alfred Wegener Institute, Helmholtz Centre for Polar and Marine Research, Bremerhaven, Germany

Correspondence: Anabel von Jackowski ajackowski@geomar.de

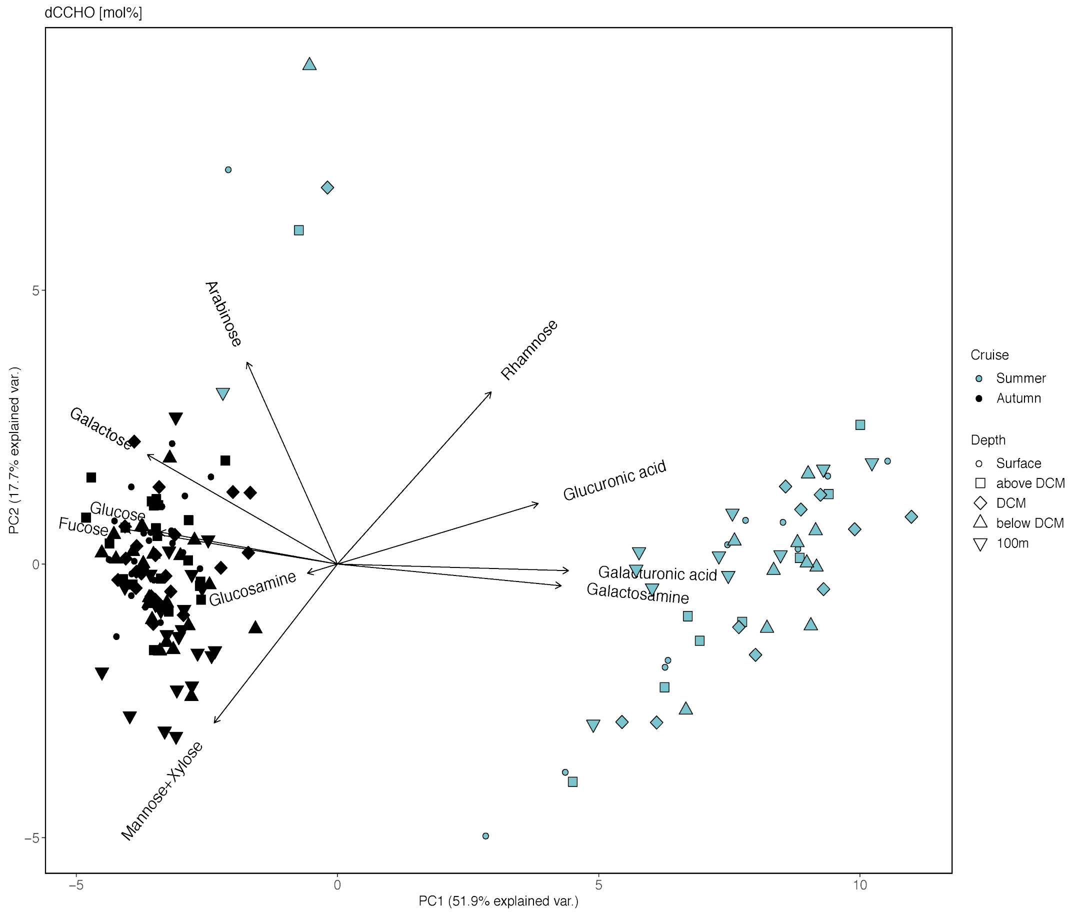

Figure S1: Relative composition (mol%) of dissolved combined carbohydrates (dCCHO) to determine a change in quality within the upper 100 m of the Fram Strait between summer and autumn. The summer samples were collected from July 16th to July 23rd, 2018 and autumn samples from September 16th to October 4th, 2018. The colour shows the two seasons: summer in blue and autumn in black. The shapes show the five depths: Surface (circle), above DCM (square), DCM (rhombus), below DCM (triangle faced up) and 100m (triangle faced down).

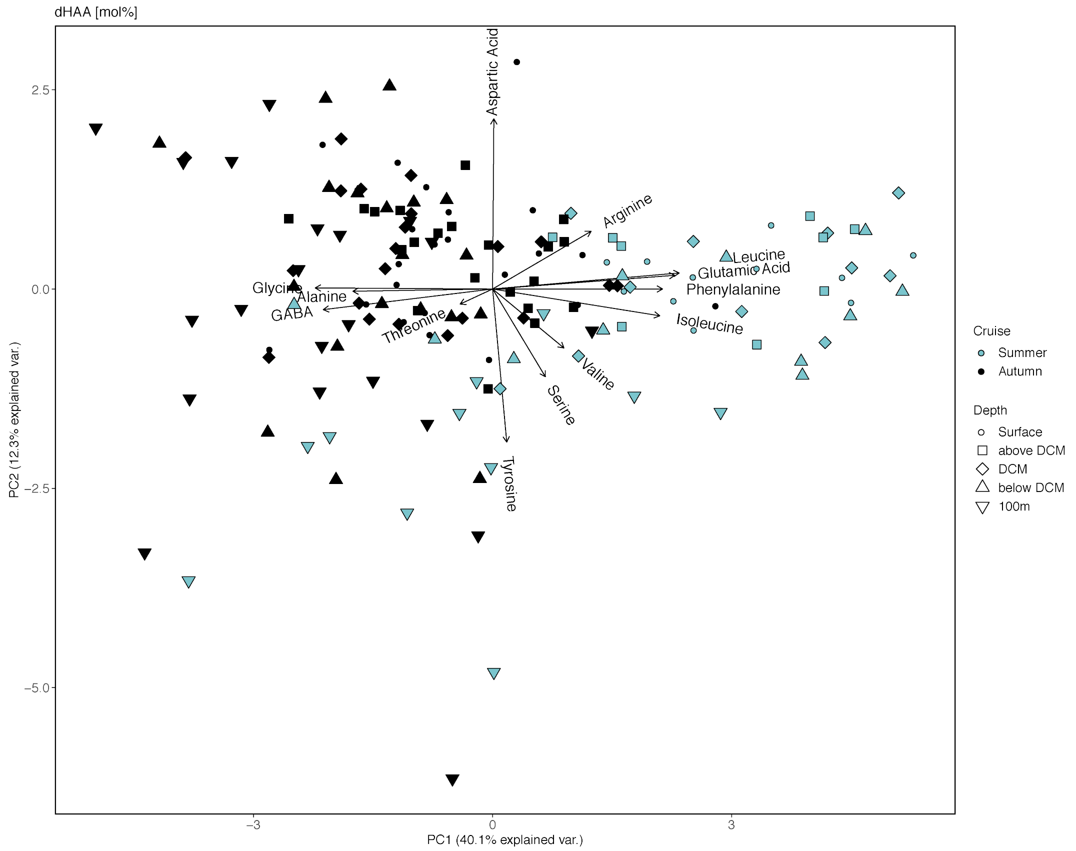

Figure S2: Relative composition (mol%) of dissolved hydrolysable amino acids (dHAA) to determine a change in quality within the upper 100 m of the Fram Strait between summer and autumn. The summer samples were collected from July 16th to July 23rd, 2018 and autumn samples from September 16th to October 4th, 2018. The colour shows the two seasons: summer in blue and autumn in black. The shapes show the five depths: Surface (circle), above DCM (square), DCM (rhombus), below DCM (triangle faced up) and 100m (triangle faced down).

Table S1: Stations within LTER HAUSGARTEN observatory and the associated CTD cast sampled in the upper 100m of the Fram Strait in the summer and autumn. The summer samples were collected with the RV Polarstern (PS114) from July 16th to July 23rd, 2018 and autumn samples with the RV Maria S. Merian (MSM77) from September 16th to October 4th, 2018. CTD dataset is available doi.pangaea.de/10.1594/PANGAEA.907467 and discrete samples are available: doi.org/10.1594/PANGAEA.915751.

Season	Station	Sampling Date (dd.mm.yyyy)	CTD ID	Latitude (degrees)	Longitude (degrees)
Summer	HG1_2	19.07.2018	PS114_20-1	79.024439	5.625398
Summer	HG4	16.07.2018	PS114_4-1	79.023712	4.332207
Summer	N3	23.07.2018	PS114_33-1	79.598585	5.166204
Summer	N4	22.07.2018	PS114_32-2	79.741002	4.52256
Summer	N5	22.07.2018	PS114_31-1	79.945191	3.200565
Summer	R1	19.07.2018	PS114_23-1	78.187393	-0.007952
Summer	R2	20.07.2018	PS114_25-2	78.83127	-0.05449
Summer	S3	17.07.2018	PS114_9-1	78.61647	5.06801
Summer	SV2	19.07.2018	PS114_16-1	78.980277	9.298997
Summer	SV3	18.07.2018	PS114_13-2	79.019027	8.007845
Summer	SV4	18.07.2018	PS114_12-1	79.011806	7.035105
Autumn	D1	30.09.2018	MSM77_44-1	78.999667	1.501167
Autumn	D2	02.10.2018	MSM77_48-1	78.8825	0.300667
Autumn	D3	02.10.2018	MSM77_47-1	78.749833	-0.883167
Autumn	D4_2	01.10.2018	MSM77_46-4	78.549167	-1.836833
Autumn	HG1	20.09.2018	MSM77_13-1	79.133	6.094167
Autumn	HG2	20.09.2018	MSM77_12-1	79.130167	4.9025
Autumn	HG3	19.09.2018	MSM77_8-1	79.108	4.6005
Autumn	HG4	17.09.2018	MSM77_4-3	79.059167	4.200167
Autumn	HG5	28.09.2018	MSM77_36-1	79.063167	3.6595
Autumn	HG6	18.09.2018	MSM77_6-1	79.06	3.5825
Autumn	HG7	28.09.2018	MSM77_37-1	79.060167	3.477167
Autumn	HG8	29.09.2018	MSM77_40-1	79.064333	3.337333
Autumn	HG9	29.09.2018	MSM77_41-1	79.133667	2.837167
Autumn	N3	04.10.2018	MSM77_54-1	79.6035	5.173
Autumn	N4	04.10.2018	MSM77_53-3	79.736333	4.485
Autumn	N5	03.10.2018	MSM77_52-1	79.938	3.194833
Autumn	NSB_1	24.09.2018	MSM77_29-1	80.3	13.999
Autumn	S3	16.09.2018	MSM77_3-1	78.616167	5.0675
Autumn	SV1	23.09.2018	MSM77_24-1	79.028333	11.086167
Autumn	SV2	23.09.2018	MSM77_22-1	78.98	9.514
Autumn	SV3	22.09.2018	MSM77_19-1	78.999833	8.25
Autumn	SV4	21.09.2018	MSM77_17-1	79.029833	6.9945

Table S2: Statistical analysis run for the study on all discrete parameters for the Fram Strait between summer and autumn. The summer samples were collected from July 16th to July 23rd, 2018 and autumn samples from September 16th to October 4th, 2018. The tests include: \textit{t}-test, a statistical mixed model, an analysis of variances (ANOVA) and multiple contrast tests (MCT). MCT we only conducted if the ANOVA was significant. For details please refer to Methods section. Abbreviations for the parameters are: dissolved organic carbon (DOC), dissolved combined carbohydrates (dCCHO), dissolved hydrolysable amino acid (dHAA), particle area of transparent exopolymer particle (TEP Area), particle area of Coomassie Blue stainable particles (CSP Area), bacterial abundance (BA), low nucleic acid bacteria (LNA), high nucleic acid bacteria (HNA) and bacterial production (BP). "AT" refers to Atlantic water and "IW" to intermediate water based on temperature-salinity characteristics.

Test	Variable	W-statistic	p-value
Mann-Whitney-test	Season:Temperature	2993	0.7836
	Season:Salinity	3554	0.02359
	Season:DOC	2969.5	0.8483
	Season:SL-DOC	4896.5	1.96E-15
	Season:dCCHO	5166.5	1.53E-15
	Season:dHAA	4657	1.73E-12
	Season:TEP Area	4035	1.30E-09
	Season: CSP Area	2844	0.1302
	Season: BA	4203	5.09E-06
	Season: HNA	4595	2.68E-09
	Season: LNA	3393	0.09071
	Season: BP	5051.5	3.83E-15

Test	Variable	F-value	p-value	Further Test
Mixed Model	Temperature.Season	13.58069	0.0003	
ANOVA	Temperature.Depth	3.3058	0.01	
	Temperature.Season*Depth	2.24719	0.1	no MCT
	Temperature.Season	12.64905	0.0005	
	Temperature.Watermass	14.71478	0.0002	
	Temperature.Season*Watermass	0.69629	0.4	no MCT
	Salinity.Season	6.9	0.01	
	Salinity.Depth	10	<0.0001	
	Salinity.Season*Depth	0.8	0.5	no MCT

Salinity.Season	5	0.03	
Salinity.Watermass	70	<0.0001	
Salinity.Season*Watermass	4	0.05	no MCT
Chl-a.Season	15.22587	0.0002	
Chl-a.Depth	32.77048	<0.0001	
Chl-a.Season*Depth	4.93302	0.001	MCT
Chl-a.Season	10.55327	0.0015	
Chl-a.WaterMass	5.86452	0.0168	
Chl-aSeason*WaterMass	39.37643	<0.0001	MCT
DOC.Season	3.333	0.1	
DOC.Depth	9.625	<0.0001	
DOC.Season*Depth	3.693	0.01	MCT
DOC. Season	2.235	0.14	
DOC.WaterMass	12.806	0.0005	
DOC.Season*WaterMass	0.633	0.4277	no MCT
SL-DOC.Season	24.5203	<0.0001	
SL-DOC.Depth	27.2973	<0.0001	
SL-DOC.Season*Depth	17.1559	<0.0001	MCT
SL-DOC. Season	83.7753	<0.0001	
SL-DOC.WaterMass	8.3643	0.0045	
SL-DOC.Season*WaterMass	6.1098	0.0147	MCT
dCCHO.Season	20.1145	<0.0001	
dCCHO.Depth	18.0504	<0.0001	
dCCHO.Season*Depth	17.429	<0.0001	MCT
dCCHO. Season	84.9255	<0.0001	
dCCHO.WaterMass	6.1177	0.0146	
dCCHO.Season*WaterMass	3.6325	0.0588	no MCT
dHAA.Season	31.2305	<0.0001	
dHAA.Depth	24.8017	<0.0001	
dHAA.Season*Depth	9.7682	<0.0001	MCT
dHAA. Season	60.2193	<0.0001	

dHAA.WaterMass	10.5641	0.0015	
dHAA.Season*WaterMass	13.3638	0.0004	MCT
TEP Area.Season	19.74817	<0.0001	
TEP Area.Depth	28.57383	<0.0001	
TEP Area.Season*Depth	15.97199	<0.0001	MCT
TEP Area. Season	64.76684	<.0001	
TEP Area.WaterMass	17.50036	0.0001	
TEP Area.Season*WaterMass	1.27261	0.2615	no MCT
CSP Area.Season	30.26717	<0.0001	
CSP Area.Depth	33.75815	<0.0001	
CSP Area.Season*Depth	3.02154	0.0206	MCT
CSP Area.Season	4.00187	0.0477	
CSP Area.Watermass	2.05573	0.1542	
CSP Area.Season*Watermass	30.40804	<0.0001	MCT
BA.Season	53.60531	<0.0001	
BA.Depth	15.26894	<0.0001	
BA.Season*Depth	1.53404	0.1961	no MCT
BA.Season	40.26859	<0.0001	
BA.Watermass	1.40656	0.2377	
BA.Season*Watermass	10.58985	0.001	MCT
HNA.Season	88.68764	<0.0001	
HNA.Depth	19.87881	<0.0001	
HNA.Season*Depth	0.60274	0.6613	no MCT
HNA.Season	63.51191	<0.0001	
HNA.Watermass	5.09151	0.0256	
HNA.Season*Watermass	6.56279	0.0115	MCT
LNA.Season	16.37995	<0.0001	
LNA.Depth	8.40553	<0.0001	
LNA.Season*Depth	3.60178	0.0081	MCT
LNA.Season	13.21119	0.0004	
LNA.Watermass	0.08646	0.7692	

	LNA.Season*Watermass	14.59077	0.0002	MCT		
	BP.Season	52.02312	<0.0001			
	BP.Depth	37.0805	<0.0001			
	BP.Season*Depth	16.92963	<0.0001	MCT		
	BP.Season	67.09755	<0.0001			
	BP.Watermass	21.63673	<0.0001			
	BP.Season*Watermass	0.26929	0.6047	no MCT		
Test	Variable	Linear Hypotheses	Estimate	Std. Error	t value	p-value
Mixed Model Multiple Contrast Test (MCT)	Chl-a.Season*Depth	Summer - Autumn, Surface	0.51555	0.31929	1.615	0.43445
		Summer - Autumn, ab.DCM	0.79334	0.28457	2.788	0.03004
		Summer - Autumn DCM	1.21241	0.36087	3.36	0.00511
		Summer - Autumn, bel.DCM	1.11827	0.3007	3.719	0.00148
		Summer - Autumn, 100m	0.14809	0.09202	1.609	0.43813
	Chla.Season*WaterMass	Summer, AW-IW	0.90862	0.17291	5.255	1.12E-06
		Autumn, AW-IW	-0.2302	0.05464	-4.213	9.13E-05
		AW, Summer-Autumn	1.05848	0.16711	6.334	6.50E-09
		IW, Summer-Autumn	-0.08035	0.07084	-1.134	0.45
	DOC.Season*Depth	Summer - Autumn, Surface	4.749	2.158	2.201	0.1378
		Summer - Autumn, ab. DCM	6.293	2.32	2.712	0.0371
		Summer - Autumn, DCM	3.512	2.158	1.627	0.4246
		Summer - Autumn, bel.DCM	0.444	2.158	0.206	0.9999
		Summer - Autumn, 100m	-4.072	2.158	-1.887	0.2686
	SL-DOC.Season*Depth	Summer - Autumn, Surface	4.9681	0.7556	6.575	0.00001
		Summer - Autumn, ab.DCM	5.6437	1.1984	4.709	0.0000326
		Summer - Autumn, DCM	4.864	1.0946	4.444	0.0000965
		Summer - Autumn, bel.DCM	3.1284	0.6632	4.717	0.0000314
		Summer - Autumn, 100m	0.6474	0.1796	3.604	0.00226
	SL-DOC.Season*WaterMass	Summer, AW-IW	1.3895	0.7174	1.937	0.10662
		Autumn, AW-IW	-0.4129	0.1256	-3.288	0.00259
		AW, Summer-Autumn	4.2131	0.5826	7.232	1.00E-10
		IW, Summer-Autumn	2.4108	0.4416	5.459	0.000000467

dCCHO.Season*Depth	Summer - Autumn, Surface	660.79	107.02	6.175	0.00001
	Summer - Autumn, ab.DCM	744.68	162.8	4.574	0.0000546
	Summer - Autumn, DCM	643.36	135.26	4.756	0.0000257
	Summer - Autumn, bel.DCM	371.03	73.08	5.077	0.00001
	Summer - Autumn, 100m	73.99	24.15	3.064	0.0132
dHAA.Season*Depth	Summer - Autumn, Surface	272.34	44.22	6.159	0.0001
	Summer - Autumn, ab.DCM	319.88	88.9	3.598	0.00231
	Summer - Autumn, DCM	274.5	71.83	3.821	0.00104
	Summer - Autumn, bel.DCM	229.25	62.57	3.664	0.00183
	Summer - Autumn, 100m	48.01	18.91	2.538	0.0601
dHAA.Season*WaterMass	Summer, AW-IW	130.477	45.41	2.873	0.009463
	Autumn, AW-IW	-39.629	9.982	-3.97	0.000237
	AW, Summer-Autumn	267.68	37.92	7.059	1.80E-10
	IW, Summer-Autumn	97.57	27	3.614	0.00086
TEP Area.Season*Depth	Summer - Autumn, Surface	117.649	18.67	6.302	0.00001
	Summer - Autumn, ab.DCM	84.47	13.909	6.073	0.00001
	Summer - Autumn, DCM	82.513	19.779	4.172	0.000289
	Summer - Autumn, bel.DCM	66.608	12.638	5.271	0.00001
	Summer - Autumn , 100m	10.332	6.017	1.717	0.367442
CSP Area.Season*Depth	Summer - Autumn, Surface	14.83	29.52	0.502	0.991337
	Summer - Autumn, ab.DCM	33.87	17.93	1.889	0.268547
	Summer - Autumn, DCM	62.84	29.07	2.162	0.15167
	Summer - Autumn, bel.DCM	107.99	24.89	4.339	0.000153
	Summer - Autumn, 100m	16.55	14	1.182	0.740625
CSP Area.Season*WaterMass	AW, Summer-Autumn	80.84	17.06	4.739	0.0000117
	IW, Summer-Autumn	-54.03	17.75	-3.044	0.0057
BA.Season*WaterMass	AW, Summer-Autumn	4.1887	0.5797	7.225	1.00E-10
	IW, Summer-Autumn	0.3908	1.0241	0.382	0.912
HNA.Season*WaterMass	Summer, AW-IW	53692	56798	0.945	0.57153
	Autumn, AW-IW	-122545	37033	-3.309	0.00239
	AW, Summer-Autumn	289058	34170	8.459	1.00E-10

	IW, Summer-Autumn	112821	60370	1.869	0.123
LNA.Season*Depth	Summer - Autumn, Surface	19178	40025	0.479	0.992885
	Summer - Autumn, ab.DCM	29527	43082	0.685	0.965169
	Summer - Autumn, DCM	50108	40025	1.252	0.690333
	Summer - Autumn, bel.DCM	151842	40025	3.794	0.001134
	Summer - Autumn, 100m	176619	40025	4.413	0.000107
LNA.Season*WaterMass	AW, Summer-Autumn	129936	26374	4.927	0.00000478
	IW, Summer-Autumn	-73118	46676	-1.566	0.224
BP.Season*Depth	Summer - Autumn, Surface	0.90683	0.11995	7.56	0.00001
	Summer - Autumn, ab.DCM	0.64977	0.14665	4.431	0.0000999
	Summer - Autumn, DCM	0.52897	0.11756	4.499	0.0000751
	Summer - Autumn, bel.DCM	0.40151	0.07939	5.058	0.00001
	Summer - Autumn, 100m	0.10697	0.03048	3.509	0.00309
